# Discrepancies in the Microstructures of Annealed Cu–Zr Bulk Alloy and Cu–Zr Alloy Films

**DOI:** 10.3390/ma12152467

**Published:** 2019-08-02

**Authors:** Haoliang Sun, Xiaoxue Huang, Xinxin Lian, Guangxin Wang

**Affiliations:** 1 School of Materials Science and Engineering, Henan University of Science and Technology, Luoyang 471003, China; 2 Collaborative Innovation Center of Nonferrous Metals Henan Province, Luoyang 471003, China

**Keywords:** Cu–Zr bulk alloys, Cu–Zr alloy films, flexible substrate, annealing, Cu particle, residual stress

## Abstract

Copper–zirconium bulk alloy and Cu–Zr alloy films are prepared by vacuum smelting and magnetron sputtering, respectively, and subsequently annealing is conducted. Results show that Cu–Zr bulk alloy and alloy films exhibit significantly different microstructure evolution behaviors after annealing due to different microstructures and residual stress states. Cu_x_Zr alloy compounds disperse at the grain boundary of Cu grains in as-cast and annealed Cu–Zr bulk alloys. However, unlike bulk alloys, a large number of polyhedral Cu particles are formed on the Cu–Zr thin films’ surface upon thermal annealing. Kinetically, the residual compressive stress in the Cu–Zr films promotes the formation of Cu particles. The influencing factors and the path for mass transport in the formation of the particles are discussed. The large-specific surface area particles/film composite structure has potential applications in Surface-Enhanced Raman Scattering, catalysis, and other fields.

## 1. Introduction

As the thickness of the film decreases to nanometer scale, the atomic diffusion and migration behaviors in thermal, electric, and stress fields become increasingly prominent during preparation and service processes [1,2,3,4,5,6]. The resistance to electric, thermal, and stress induced atomic migrations in Cu thin films are of great importance for the reliability of electronic devices. It was found that alloying with elements with high melting points, such as Zr, Cr, and Mo, could dramatically improve the resistance of Cu to electric and stress induced migrations, and thus enhance the service life of Cu lines and electric devices [7,8]. Hillocks are commonly produced on Cu and other metal films [9,10,11,12,13,14] on Si substrates upon annealing. However, less attention has been paid to the evolution of microstructures and physical properties of electric devices on flexible polyimide (PI) substrates. Since the thermal expansion coefficients of metal thin films are larger than those of Si substrates, but less than those of PI substrates, the residual stress and microstructure evolution in metal films on two kinds of different substrates might be distinct. 

When the geometrical dimension of the material is reduced to a critical dimension in at least a one-dimensional direction, various physical mechanisms of the materials will “feel” the effect of the surface and interface [15]. Previous research [15,16,17,18,19] has shown that macroscopic bulk materials and corresponding nano-film materials usually exhibit different properties. Ghidelli et al. reported extrinsic mechanical size effects in thin Zr–Ni metallic glass films [20] and the yield stress increased with decreasing specimen size up to the ideal yield strength [21]. Certain components of Cu–Zr bulk alloys tend to form amorphous structures, but study on the thermal stability of the Zr–Cu films (from 13.4 to 85.0 at.% Cu) indicated that some films are in fact composed of nanometer-size crystallized domains, and only the Zr-48.3 at.% Cu film is amorphous [22]. Khobragade et al. [23] found that the addition of 0.75 at.% Zr results in stability of the nanocrysalline state of copper at higher temperatures. In this paper, a comparative study of the microstructure evolution of Cu–Zr bulks and Cu–Zr alloyed thin films on flexible PI substrates are investigated.

## 2. Experimental Details

Cu-1.1 at.% Zr bulks were prepared by vacuum smelting and some samples were processed into 10 × 10 × 10 mm cubes for vacuum annealing. Cu–Zr alloyed thin films and pure Cu thin films were deposited on the polyimide (PI) substrates with a thickness of 125 μm by using DC-magnetron sputtering. The substrates were ultrasonically cleaned for 15 min before insertion into the vacuum chamber. Pure Cu target (purity 99.99 wt.%) and Cu–Zr composite targets composed of the pure Cu target and Zr slices (purity 99.95 wt.%) were used to prepare pure Cu films and Cu–Zr alloy films, respectively. The composition of the Cu–Zr bulks and alloy films was measured by energy dispersive spectrometer (EDS). The base pressure of the chamber was evacuated to 2 × 10^−4^ Pa. The deposition was conducted under a pressure of 0.3 Pa with an Ar gas flow of 20 sccm. The sputtering power was 100 W and the distance of the substrate target was 65 mm. In situ thermal annealing was conducted in the temperature range of 180~450 °C in a vacuum chamber for 60 min and furnace cooling was held for all the annealed samples. The composition of the Cu–Zr bulk alloy and Cu–Zr alloy films with different thickness are shown in Table 1.

X-ray diffraction (XRD, 7000S X-ray Diffractometer, SHIMADZU LIMITED, Kyoto, Japan) (Cu K-alpha), field emission scanning electron microscope (FE-SEM, JSM 6700F, JSM 7100, JEOL Ltd., Tokyo, Japan, high resolution transmission electron microscope (HRTEM, JSM 2100F, JEOL Ltd., Tokyo, Japan), and energy dispersive spectrometry (EDS) were used to characterize the surface morphology, the crystal structure, and the composition of the Cu–Zr alloy films. 

During the preparation of Cu–Zr bulk alloy and Cu–Zr alloy films, trace oxygen in the vacuum readily reacted with Zr or adsorbed on the surface of samples. Additionally, C and O also easily adsorbed on the surface of samples during vacuum annealing, which may have resulted in the existence of C and O in the EDS results.

Film thickness was measured from the cross-section of FE-SEM images. The residual stress of the thin films was measured by XRD implemented in position-sensitive proportional counter (PSPC) and based on the sin^2^*Ψ* method.

## 3. Results and Discussion

### 3.1. Microstructural Evolution in Annealed Cu–Zr Bulk Alloys

**Figure 1:**
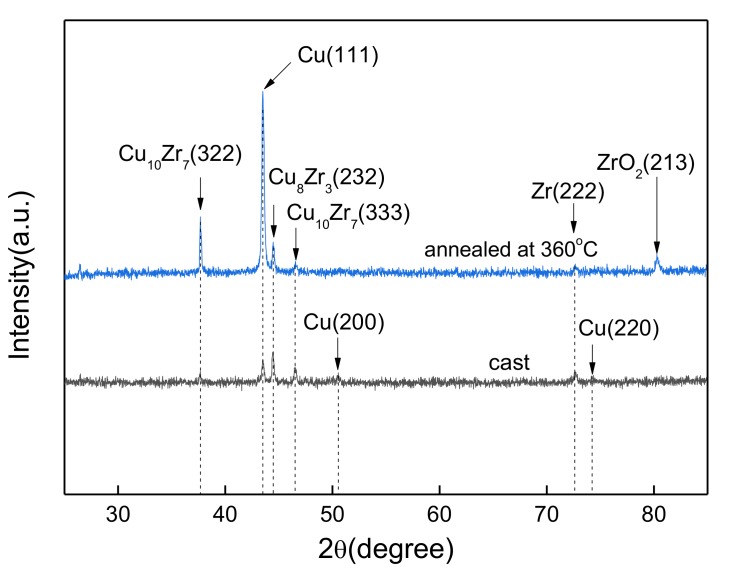
X-ray diffraction (XRD) patterns of as-cast and annealed Cu-1.1%Zr bulk alloys.

Figure 1 shows the XRD patterns of as-cast and annealed Cu–Zr bulk alloys. It can be seen from Figure 1 that the diffraction peaks of the Cu_10_Zr_7_ (333) and Cu_8_Zr_3_ (232) compounds can also be observed on the XRD pattern of as-cast alloys, besides the obvious diffraction peaks of Cu (111), which indicate that the Cu_x_Zr alloy compounds were formed during the melting process of Cu–Zr bulk alloys. Compared with as-cast alloys, the intensity of diffraction peaks of the Cu–Zr bulk alloys annealed at 360 °C increased significantly, indicating that the grain size of the alloys grew gradually during annealing. In addition, weak Zr (222) and ZrO_2_ (213) diffraction peaks can also be observed in the XRD spectra of annealed Cu–Zr bulk alloys, which can be attributed to the fact that Cu and Zr are almost insoluble at room temperature, and the supersaturated fine Zr grains dispersed at the Cu grain boundaries. The appearance of ZrO_2_ (213) implies that the fine Zr grains on the surface of the alloy easily adsorb oxygen to form ZrO_2_ during preparation, annealing, or storage at room temperature.

**Figure 2:**
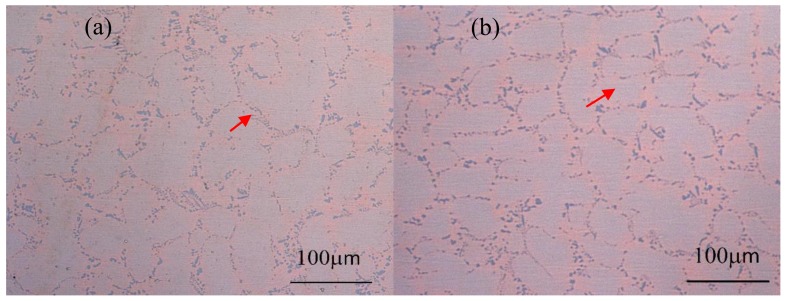

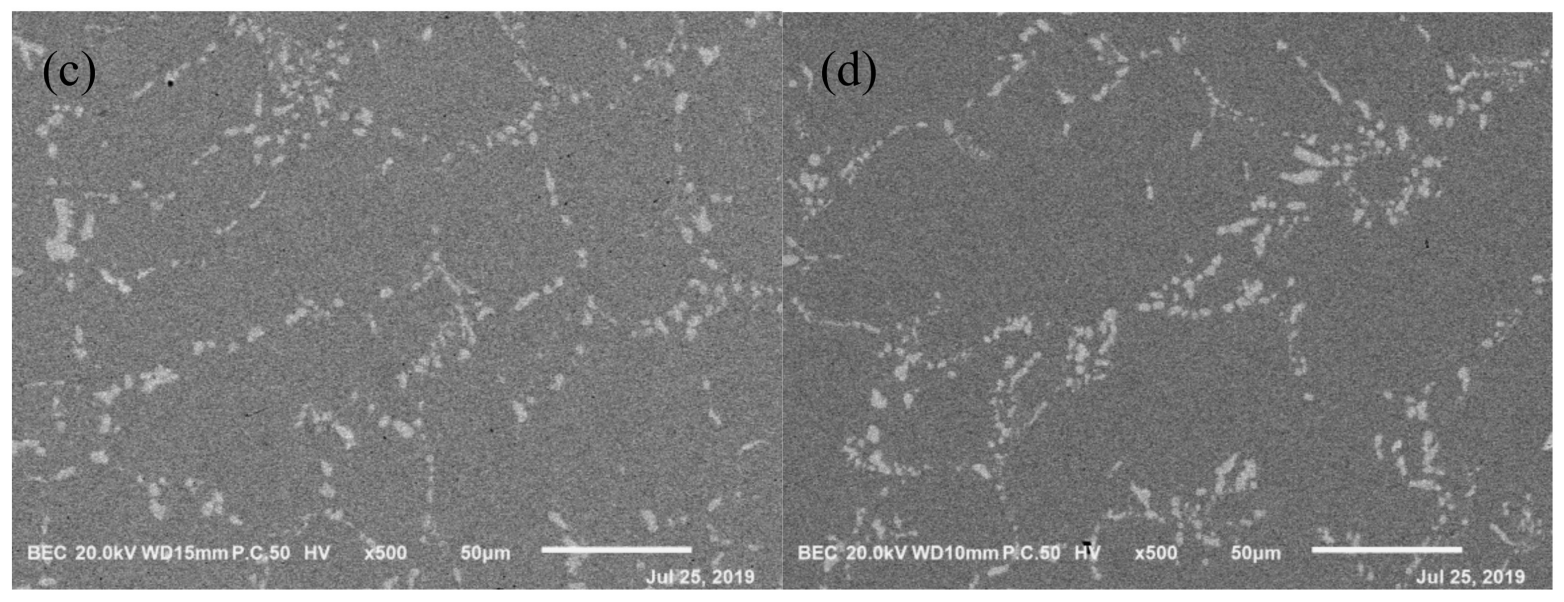
Metallographic of (**a**) as-cast and (**b**) annealed Cu–Zr bulk alloys without corrosion and field emission scanning electron microscope (FE-SEM) images of (**c**) as-cast and (**d**) 360 °C annealed Cu–Zr bulk alloy.

Figure 2 shows a metallographic of as-cast and annealed Cu-1.1%Zr bulk alloys before corrosion. Compared with Figure 2a,b, it can be clearly seen that a large number of Cu_x_Zr alloy compounds, including Cu_10_Zr_7_ and Cu_8_Zr_3_, were dispersed at the grain boundaries of Cu grains in the as-cast and Cu–Zr bulk alloys annealed at 360 °C. As shown in the red front of Figure 2, after annealing at 360 °C, the size of the Cu_x_Zr alloy compounds increased gradually due to the more active atom diffusion during annealing. The Cu_x_Zr alloy compounds dispersed at the grain boundaries of Cu grains inhibited the growth of Cu grains to a certain extent during annealing, resulting in little change in the average grain size of Cu grains.

The SEM images of the Cu–Zr bulk alloy are shown in Figure 2c,d. Cu–Zr alloy compounds grew dispersed in Cu grain boundaries in the (c) as-cast and (d) 360 °C annealed Cu–Zr bulk alloys. The size of Cu–Zr alloy compounds increased slightly after annealing, which is in agreement with the metallographic picture.

### 3.2. Microstructure in As-deposited and Annealed Cu–Zr Alloy Films

**Figure 3:**
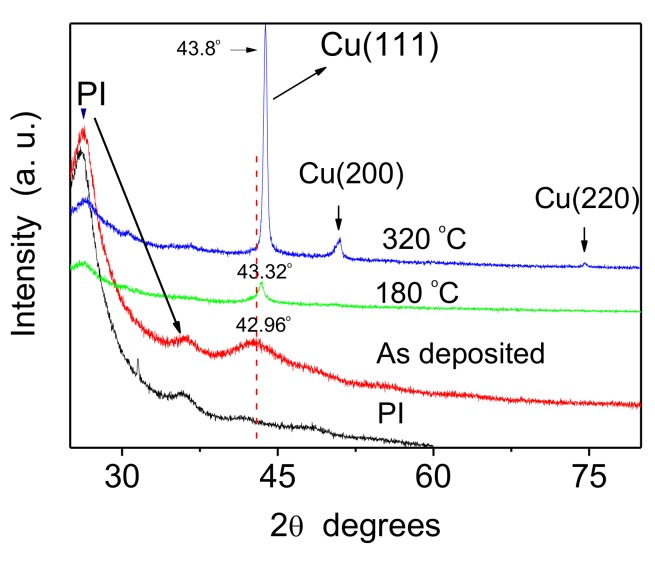
XRD patterns of the polyimide (PI) substrate, and the deposited and annealed Cu-17.1%Zr alloy films.

Cu–Zr alloyed thin films with different Zr contents were prepared with thicknesses ranging from 50 nm to 420 nm, and EDS spectra showed that the Zr content ranged from 7.3 to 17.1 at.%. Figure 3 shows the XRD patterns of the as-deposited and annealed Cu-17.1% Zr alloy films. The XRD pattern of the as-deposited films exhibits a weak diffraction peak, indicating a tiny grain size or amorphous structure in the alloy films [22]. If the Zr content is further increased, it is possible to form amorphous Cu–Zr alloyed films. No diffraction peaks of elemental Zr and Cu_x_Zr compounds were observed which is different from those in Cu–Zr bulk alloys, as shown in Figure 1. The reason is that Cu and Zr are immiscible in the alloyed film at room temperature, and the fine Zr grains dispersed at Cu grain boundaries. However, thermal annealing affects the microstructure in the alloyed thin films greatly, but only Cu diffraction peaks appear in the XRD patterns. The preferential growth of Cu (111) grains is visible, and the XRD intensity of Cu (111) diffraction peak gradually increased with annealing temperature, as a result of increased grain size in the alloyed thin films. The Cu (200) and Cu (220) diffraction peaks became weak when the annealing temperature increased up to 320 °C.

**Figure 4:**
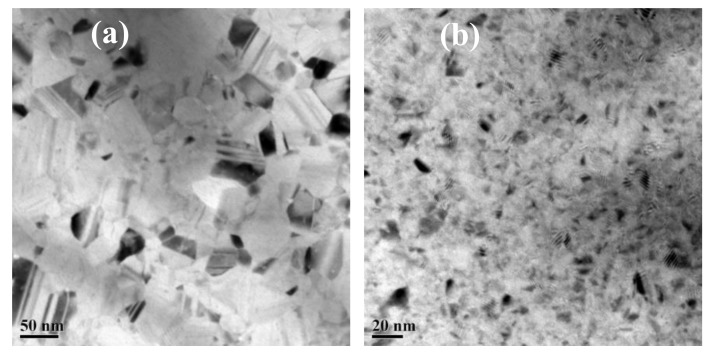
Bright-field TEM images of (**a**) the as-deposited Cu and (**b**) Cu-7.3%Zr thin films.

Figure 4a,b shows the TEM results of the as-deposited Cu and Cu-7.3%Zr alloy films of 50 nm thickness. The average grain size in the pure Cu thin films was 50~60 nm with clear grain boundaries, as shown in Figure 4a. However, the grains in the Cu–Zr alloy thin films were very tiny, only 10~20 nm, as shown in Figure 4b. This may be due to the large amount of tiny Zr grains dispersed in the copper grain boundaries, which inhibited the growth of Cu grains. 

**Figure 5:**
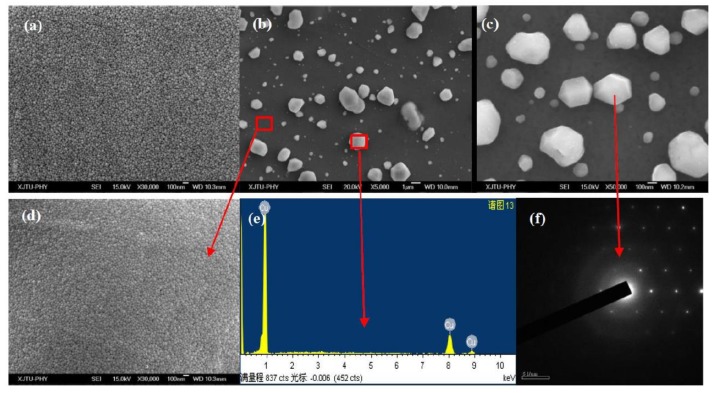
Field emission scanning electron microscope (FE-SEM) images of the surface morphology of the as-deposited and annealed Cu–Zr alloy films, and the selected area electron diffraction (SAED) patterns of a Cu particle. (**a**) Cu-7.3%Zr deposited film, (**b**) Cu-7.3%Zr film annealed at 300 °C, (**c**) Cu-17.1%Zr film annealed at 300 °C, (**d**) the high magnification image of the surface morphology in the nearby regions without self-formation Cu particles in (**b**), (**e**) the energy dispersive spectrometry (EDS) pattern of a self-formation faceted particle in (**b**), (**f**) the SAED patterns of the self-formation faceted particles in (**c**).

Figure 5 shows the surface morphology of the as-deposited and annealed Cu–Zr alloy films with different Zr contents, as well as the selected area electron diffraction (SAED) patterns of the self-formation faceted particles. The particles of the as-deposited films’ surface were very fine and uniform, as shown in Figure 5a. In contrast to the flat surface of as-deposited alloy films, a large number of polyhedral particles emerged on the surface of the annealed Cu–Zr films. It should be pointed out that some particles were faceted and regular, as shown in Figure 5b,c. The EDS results indicate that the composition of the regular particles was pure Cu, as shown in Figure 5e, which is different from the Cu-rich particles obtained in previous studies [8]. The average sizes of Cu particles in Figure 5b,c are 369 and 173 nm, respectively. The results in the present work the earlier studies [24,25] demonstrate that the size of Cu particles can be controlled by changing the film’s composition and annealing condition. 

The SAED pattern in Figure 5f shows that the faceted Cu particles formed on the Cu–Zr alloy film were single crystals. As shown in Figure 5b, some adjacent particles gradually grew into large polycrystalline particles. Figure 5d shows the high-resolution image of surface morphology in the regions without the self-formed Cu particles in Figure 5b. The particles in the nearby regions are very fine and uniform, and the size is slightly larger than that of as-deposited alloy films in Figure 5a.

Comparing Figure 2 and Figure 5, it can be clearly seen that Cu–Zr bulk alloy and alloy films exhibited significantly different microstructure evolution behaviors after annealing. Many Cu_x_Zr alloy compounds dispersed at the grain boundary of Cu grains in annealed Cu–Zr bulk alloys. However, unlike bulks, numerous polyhedral Cu particles of sub-micron were formed on the Cu–Zr thin films’ surface upon thermal annealing. This difference is due to the obvious difference in the microstructure and residual stress state caused by different preparation methods [15,19], which leads to different microstructural evolution behaviors between bulk materials and thin film materials during annealing. Cu–Zr bulk materials are stable materials obtained by vacuum melting. During melting and the subsequent pouring process, the Cu and Zr atoms in the high temperature environment are easily diffused to form Cu_x_Zr alloy compounds, which grow further during annealing. Thin film materials are metastable materials obtained by magnetron sputtering. In the process of thin film deposition, it is difficult for Cu and Zr atoms to diffuse into Cu_x_Zr alloy compounds in a near room temperature environment. Therefore, no alloy compound was found in XRD phase analysis and SEM surface morphology observation. Kinetically, the relaxation of the compressive residual stress and thermal stress in the Cu–Zr alloy films resulted in the faceted Cu particles forming on the alloy film surface [24,25].

### 3.3. The Main Influencing Factors of Self-formed Cu Particles

**Figure 6:**
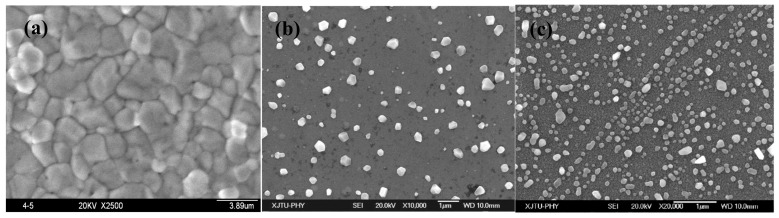
The surface morphologies of Cu–Zr films after annealing at 300 °C with different compositions, (**a**) Cu-0%Zr, (**b**) Cu-7.3%Zr, and (**c**) Cu-12.3%Zr.

Figure 6 shows the surface morphologies of 420 nm thick alloy films with different Zr contents after annealing at 300 °C. As displayed in Figure 6a, the grains in the Cu films became larger after annealing, and some grains protruded from the surface. It can be seen in Figure 6b,c that a lot of polyhedral particles appeared on the annealed Cu–Zr films, and they were significantly different from the Al hillocks protruding from the annealed Al films [11], as well as the Cu grain growth protruding from the annealed Cu films in Figure 6a. The mean sizes of Cu particles in Figure 6b,c are 312 and 95 nm, respectively. For the same film thickness and annealing process, the number of particles increases, but the particle size decreases with increasing Zr content. The higher Zr content in the Cu–Zr alloy films can inhibit the diffusion of Cu atoms more obviously and the growth of Cu particles. At the same time, a high Zr content may lead to more triple line junctions appearing near the film surface, which is conducive to the accumulation and nucleation of Cu atoms.

**Figure 7:**
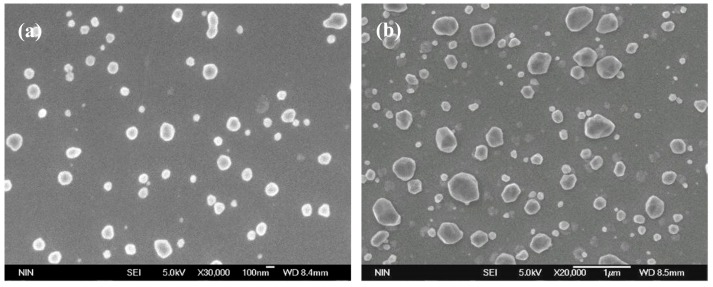
Surface morphologies of 225 nm thick Cu-17.1%Zr films after annealing at different temperatures: (**a**) 180 °C, (**b**) 320 °C.

Figure 7 shows the surface morphologies of 225 nm thick Cu-17.1%Zr films after annealing. It can be seen in Figure 7a,b that both the particle size and the particle number increased with annealing temperature for the same film thickness and composition. The mean sizes of Cu particles in Figure 7a,b are 99 and 206 nm, respectively.

**Figure 8:**
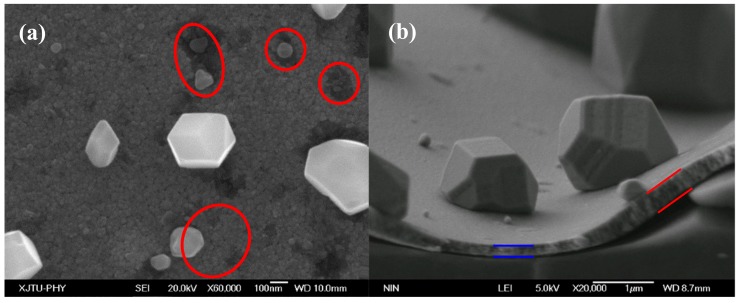
The surface and cross-sectional morphologies of the annealed Cu–Zr films. (**a**), the Cu atoms consumed, leaving some “shallow pits” on the alloy film surface, (**b**) the cross-sectional morphology of alloy films annealed at 450 °C showing inhomogeneous film thickness due to formation of Cu particles.

Figure 8 shows the surface and cross-sectional morphologies of the annealed Cu–Zr film. The formation of a large number of polyhedral pure Cu particles inevitably consumes a lot of Cu atoms surrounding the nucleation sites. As a result, shallow pits emerged on the alloy films, as indicated by the red circle area in Figure 8a, and they connected with each other, gradually accompanied by the growth of Cu particles. Thus, the film became thinner with the growth of many particles, as confirmed by the cross-sectional SEM image in Figure 8b. The thickness of Cu–Zr films underneath self-formed Cu particles was thinner than that away from Cu particles, as shown in Figure 8b. Since the interface was almost in perfect coincidence with the film surface, the surface diffusion of the Cu atoms seems to be the dominant mass transport mechanism of Cu particle growth.

**Figure 9:**
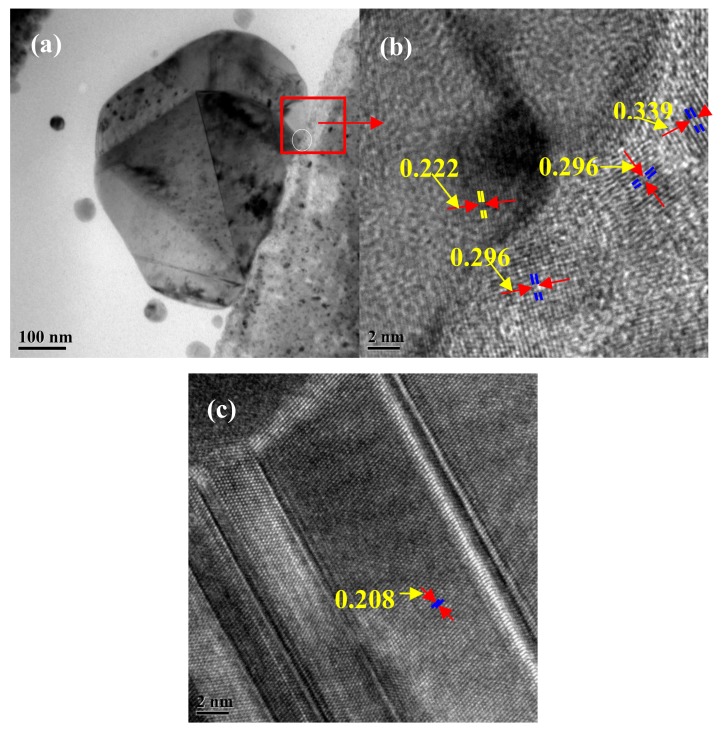
Bright-field TEM images of (**a**) a large Cu particle growth on the surface of the alloy film, (**b**) the high magnification image of (**a**), and (**c**) a normal grain of a pure Cu film.

Figure 9 shows the bright-field high resolution transmission electron microscope (HRTEM) images of the interface between the Cu particle and the alloy films. As displayed in Figure 9a, a large Cu particle grew on the alloy film and was well bonded with the film, with clear boundary. It can be seen from the high-resolution image of the interface that the self-formed Cu particles were coherent with the film (Figure 9b). This is completely different from the Al hillocks in the pure Al films [11]. It can be seen from Figure 8 and Figure 9 that the Cu particles nucleated and grew on the film surface, while the Al hillock grew inside pure Al film [11]—the nucleation positions of them were completely different. Figure 9c shows the bright-field TEM images of a normal grain in Cu film. As compared to Figure 9c, it can be found from Figure 9b that the supersaturated Zr in the alloy film resulted in lattice distortion in the alloy films. Thus, the release of distortion energy during annealing is one of the driving forces to form Cu particles.

**Figure 10:**
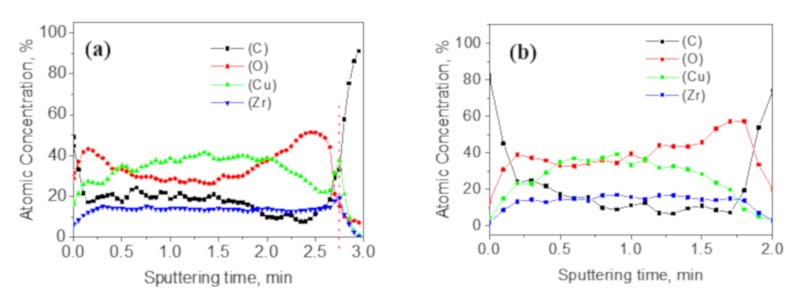
Auger Electron Spectroscopy (AES) diagrams of a 50 nm Cu-7.3%Zr alloy film: (**a**) as deposited, (**b**) annealed films.

Figure 10 shows the Auger Electron Spectroscopy (AES) diagrams of the 50 nm Cu-7.3%Zr alloy film before and after annealing. The sputtering area was 2 × 2 mm, and the sputtering rate was about 35 nm/min. For the as-deposited and annealed alloy films, the time required for sputtering down to the film-substrate interface was about 2.75 and 2 min, respectively, that is, the thickness of the alloy film was reduced by 22 nm after annealing. This implies that surface diffusion mainly occurs during the particle growth.

### 3.4. Residual Stress in Cu–Zr Alloy Films

**Figure 11:**
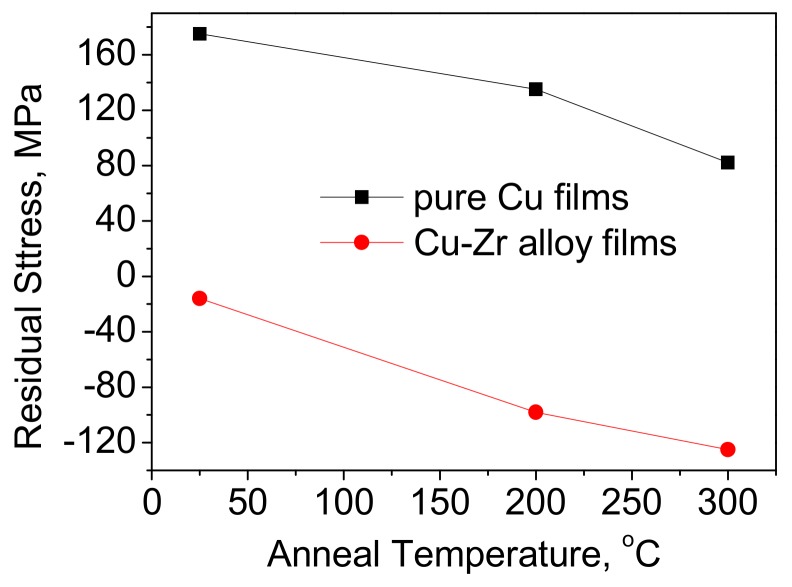
Residual stress as a function of annealing temperature for both pure Cu and Cu-7.3%Zr films.

Figure 11 shows the residual stresses results of annealed Cu films and Cu-7.3%Zr alloy films. The Cu film exhibited a tensile stress, but this was compressive in the Cu-7.3%Zr alloy film. As the annealing temperature increased from room temperature up to 300 °C, the residual stress in the Cu film was reduced from 175 MPa down to 82 MPa. However, the stress in Cu-7.3%Zr alloy film increased from −16 MPa up to −125 MPa after annealing at 300 °C. 

It has been suggested [26] that the formation of metal hillocks is due to thermal-induced stress relaxation. For the Cu–Zr alloy films in this work, Zr atoms or tiny Zr grains distributed at the grain boundary of Cu played a pinning effect and could inhibit grain boundary diffusion. Therefore, residual compressive stress will be released mainly through the formation of particles. Cu particles are preferentially formed at scratches and grooves on films, indicating that residual stress is an important driving force for the formation of particles [25]. Thermal annealing can activate the release of residual stress in thin films, and it can also induce thermal stress due to the mismatch of thermal expansion coefficients between metal films and soft PI substrates. The thermal expansion (CTE) of the PI substrates was 29.5 × 10^−6^ °C^−1^, and the CTE of Cu–Zr alloy films (*α_f_*) was evaluated according to the mixture rule, supposing that Cu and Zr atoms were uniformly mixed together in the film:

(1) $$\alpha_{f}=f\alpha_{1}+(1-f)\alpha_{2}$$

where *α*_1_ is 6.9 × 10^−6^ °C^−1^ for CTE of pure Zr, *α*_2_ is 16.5 × 10^−6^ °C^–1^ for CTE of Cu, and *f* is the volume fraction of Zr atoms and can be calculated from the atomic percent of Zr in the film. The elastic modulus of a Cu–Zr alloy *E_f_* was also evaluated by the mixture rule:

(2) $$E_{f}=fE_{1}+(1-f)E_{2}$$

where *E*_1_ is 97 GPa of pure Zr, *E*_2_ is 130 GPa of pure Cu, and *f* is the volume fraction of Zr in Cu–Zr film. Furthermore, the thermal stress was calculated according to the Stony Equation (3) [27]:

(3) σth=Ef1-υfαf-αsT1-T0

where *T*_0_ and *T*_1_ denote the room and annealing temperature, respectively, and *α_f_* and *α_s_* denote the CTE of film and the CTE of substrate, respectively, *E_f_* is the elastic modulus, and Posson’s ratio ν_f_ is taken as 0.34 [28] for the Cu–Zr/PI system. 

**Figure 12:**
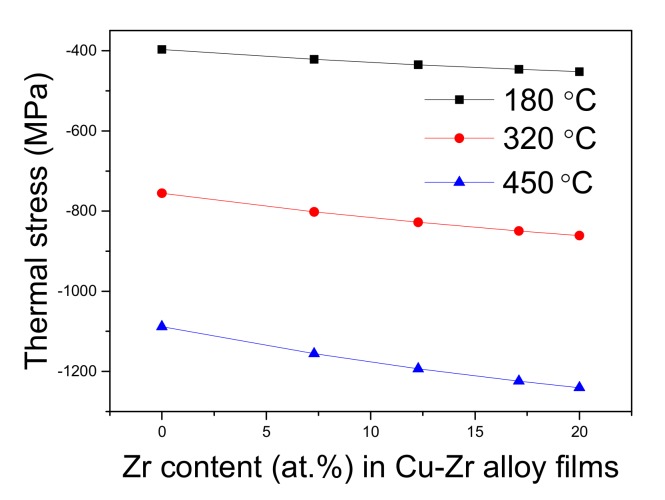
Thermal stress as a function of Zr content for Cu–Zr films annealed at three different temperatures.

Figure 12 shows the calculated thermal stress of alloy films annealed at different temperatures. A compressive thermal stress existed in the thin films because the CTE of the PI substrate was larger than that of Cu–Zr films, and the thermal stress was in the range of −397 to −1240 MPa, which was large enough to drive the formation of Cu particles on Cu–Zr film. The thermal stress increased with Zr content, and more particles emerged on the films of higher Zr contents (Figure 6) and annealed at higher temperatures (Figure 7).

### 3.5. Formation Mechanism of Faceted Cu Particles on Cu–Zr Alloy Films

**Figure 13:**
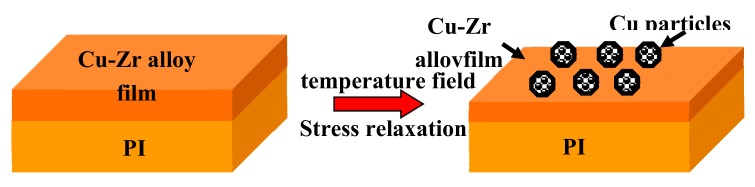
Schematic diagram of Cu particle formation on the annealed Cu–Zr alloy film’s surface.

Figure 13 shows a schematic diagram of particle formation. Surface diffusion plays an important role on the growth of Cu particles. Figure 8 and Figure 9 display the inhomogeneous film thickness and the perfect interface between Cu particles and film. The particle size decreased with increasing Zr content (Figure 6). This was probably associated with the distribution of Zr atoms. More and more Zr atoms or tiny particles were segregated at Cu grain boundaries, inhibiting Cu diffusion and the growth of Cu particles. Moreover, lattice distortion increased with Zr content, and interface energy and stress energy were enhanced, which is favorable for the formation of more small particles. Furthermore, particles seemed to form at different times during annealing. As the annealing temperature was elevated, the mean particle size and number increased, because of more active Cu atomic diffusion. Meanwhile, the stress in the alloy film was further relaxed, which also inevitably leads to Cu particle growth and the emergence of new particles.

Many studies were carried out to understand the mechanism of the hillock formation. Grain boundary diffusion [12], interfacial diffusion [29], and creep-controlled diffusion [30] have been proposed to understand the formation mechanism of hillocks. Hwang [31] and Berla [32] provided models for hillock growth based on plastic deformation respectively.

Based on the experimental results, it was illustrated that the formation mechanisms of faceted Cu particles on the annealed surface Cu–Zr alloy films surface is significantly different from that of the hillocks observed in Al and Cu films on Si substrates. The Cu particles grow by mass transportation along surfaces and grain boundaries driven by the relaxation of residual compressive stress, distortion energy, and thermal stress [33]. First, Cu atoms are aggregated to form fine clusters, and then some clusters grow up to be small Cu particles at triple grain boundaries and voids on the Cu–Zr alloy film. The mass transfer during the growth of Cu particles is accomplished by surface diffusion. 

## 4. Conclusions

Cu–Zr bulk alloy and thin films exhibit significantly different microstructure evolution behaviors after annealing. The Cu_x_Zr alloy compounds dispersed at the grain boundary of Cu grains in the as-cast and annealed Cu–Zr bulk alloys. However, in contrast to the flat surface of as-deposited alloy films, a large number of polyhedral particles were formed on the surface of annealed Cu–Zr films, which can be ascribed to the obvious difference in the microstructures and residual stress states caused by different preparation methods. Kinetically, the relaxation of residual compressive stress, distortion energy, and thermal stress results in the formation of regular faceted Cu particles on the film surface, in which the surface diffusion is the dominant mass transport mechanism. The particle size and distribution are dependent on the Zr content and annealing temperature. 

## Figures and Tables

**Table 1:** Composition of Cu–Zr bulk alloy and Cu–Zr alloy films with different thickness.

Sample	Film Thickness (nm)	Zr Content (Atomic Atom%)
Cu–Zr bulk alloy		1.1
Cu–Zr alloy thin film	50	7.3
	225	17.1
	420	0
		7.3
		12.3
